# Post-traumatic growth experience of first-line emergency nurses infected with COVID-19 during the epidemic period—A qualitative study in Shanghai, China

**DOI:** 10.3389/fpubh.2022.1015316

**Published:** 2022-10-12

**Authors:** Jinxia Jiang, Peng Han, Xiangdong Huang, Yue Liu, Haiyan Shao, Li Zeng, Xia Duan

**Affiliations:** ^1^Emergency Department, Shanghai Tenth People's Hospital, School of Medicine, Tongji University, Shanghai, China; ^2^Department of Nursing, Tongji Hospital, School of Medicine, Tongji University, Shanghai, China; ^3^Nursing Department, Shanghai First Maternity and Infant Hospital, School of Medicine, Tongji University, Shanghai, China

**Keywords:** post-traumatic growth, emergency nurses, COVID-19, psychological experience, qualitative study

## Abstract

**Background:**

In March 2022, Shanghai, China, was hit by a severe wave of SARS-CoV-2 transmission caused by the Omicron variant strain. The medical staff was greatly infected during this period, which posed a traumatic event for them. Meanwhile, they also experience post-traumatic growth under introspection and positive change. However, the psychological coping and growth after infection with COVID-19 among medical staff have rarely been investigated.

**Objectives:**

To explore the process and influencing factors of post-traumatic growth among emergency nurses infected with coronavirus disease (COVID-19) so as to provide a new perspective and theoretical basis for psychological rehabilitation or intervention for medical staff who experienced traumatic events.

**Methods:**

The study used a qualitative design based on the phenomenological approach. A purposive sampling method was used to explore the subjective feelings and post-traumatic growth among 13 first-line emergency nurses infected with COVID-19 in Shanghai, China. Semi-structured face-to-face interviews were conducted in June 2022. A Seven-step Colaizzi process was used for data analysis.

**Results:**

Themes were described and extracted from the experience and insights at different stages during the fight against the virus. Three main themes, i.e., stress period, adjustment period, and growth period, as well as several sub-themes, were identified.

**Conclusion:**

First-line emergency nurses infected with COVID-19 are a sensitive group that should be given more attention. Investigating how they achieve psychological adjustment and growth in the case of severe trauma can provide valuable references for nursing management and education in the future. Society, hospital and nursing managers should pay more attention to the PTG of nurses and establish supportive PTG strategies, which will benefit the retention rate and career development of nurses.

## Introduction

The World Health Organization (WHO) proclaimed the outbreak of the severe acute respiratory syndrome coronavirus 2 (SARS-CoV-2) that caused the 2019 coronavirus disease (COVID-19) as the Public Health Emergency of International Concern (PHEIC), characterizing it as a pandemic (1). The virus spreads mainly through saliva droplets, nose droplets from an infected person who coughs or sneezes, or airborne aerosols (2). On November 9, 2021, a novel Coronavirus B.1.1.529 variant was first detected from case samples in South Africa. Within just 2 weeks, the mutant strain has become the absolute dominant strain in Gauteng Province, South Africa, with rapid growth of COVID-19 cases. On November 26, 2021, it was defined as the fifth Variant of Concern (VOC) and was named Omicron by WHO (3). Omicron continued to mutate, and recently SARS-CoV-2 Omicron Subvariants BA.4 and BA.5 have been identified (4). Since March 2022, Shanghai, China has experienced a severe wave of SARS-CoV-2 transmission caused by the Omicron variant strain. During the time period between March 1, 2022 and June 31, 2022, a novel coronavirus infected nearly 6,50,000 people in Shanghai, with more than 58,000 confirmed local cases and 5,91,000 asymptomatic infections, including many medical staff (5).

Chinese and Shanghai governments took the spread of COVID-19 very seriously, as they did at the beginning of the outbreak in Wuhan. The government had mobilized massive medical resources to set up fever clinics, special isolation wards, hospitals for severe patients and temporary shelter hospitals (mobile cabin hospitals). More than 100 designated hospitals and mobile cabin hospitals have been added to accommodate all types of infected patients and asymptomatic infected patients (6). Tens of thousands of medical staff had been involved in treating those diagnosed with the virus (7). During the epidemic period, the treatment activities of first-line emergency medical staff included close contact with sources of infection, heavy treatment tasks, and strict protection. The occupational risks they faced were also characterized by high infection rates (8). Globally, around 14% of COVID-19 cases reported to WHO were health workers, while in some countries, this rate was as high as 35% (9). In China, the education level of nurses is mainly from junior college to undergraduate, and the study of infectious disease prevention is essential. The government attached great importance to protecting healthcare workers from infection. However, under strict protection, there were still staff members who were unfortunately infected. For those infected with COVID-19, this presented a traumatic experience. In addition to dealing with compromised physical health, psychological trauma should not be ignored.

Trauma refers to the acute damage caused by the external environment to the body tissues and organs and the patient's psychology. The most common factors include mechanical trauma, chemical trauma and psychological trauma. In psychiatry, psychological trauma is defined as “events beyond the ordinary experience,” i.e., the occurrence of traumatic events is sudden and irresistible, which makes people's psychological state deviate from the normal everyday state (10). The experience of trauma actually leads to deep psychological injury at an unconscious level that entails loss of control, language, power, and self. Trauma is a wound that “cries out,” a silent wound that is articulated through re-enactments. As a result, traumatized individuals are vulnerable to repeating past traumas and remain in a crisis without being able to regain control over their current lives (11). More than 70% of the global adult population experienced at least one traumatic event in their lifetime, while 31% experienced four or more (12). Emergency nurses are at high risk of trauma due to the serious condition of patients, high work intensity, interpersonal pressure, violence and other work related risks. Global public health events such as COVID-19 pose huge occupational risks to nurses, especially those working on the first line, who are at great risk of infection. Even in society, infected people will be stigmatized by some media and the public, they will feel ostracized and ashamed, and medical staff are no exception (13). Having experienced working during the COVID-19 pandemic, many front-line personnel had sacrificed their own wellbeing, which could be even more traumatic and cause increasing psychological distress when they were infected with the novel coronavirus (14).

Generally, mild psychological trauma can be self-healing, while the trauma of a larger degree may cause anxiety, depression, and in severe cases, even Post-Traumatic Stress Disorder (PTSD), thus putting these individuals at a high risk of Secondary Traumatic Stress (STS) and bringing more severe challenges to their mental health (15). Previous studies have found that the incidence of depressive symptoms among people diagnosed with COVID-19 is as high as 29.2%, which is significantly higher than in the general public (16). According to Missouridou's research, nurses who have experienced traumatic events were more prone to develop compassion fatigue during the COVID-19 pandemic (17). The inability to cope with the impact of trauma may limit nurses' ability to interact in a meaningful and safe way with patients and their families (11). Yet, in recent years, some scholars have tried to understand people's subjective feelings other than STS from a positive perspective. Positive psychological changes that occur after an individual experiences traumatic events are known as Post-Traumatic Growth (PTG) (18), i.e., one has the ability to grow as a result of trauma (19). After working on the first line, infected nurses may experience some positive changes due to the COVID-19 awareness and coping experience gained on the job. PTG helps them reflect on their experience, which is beneficial to their career growth and general satisfaction with life (20).

The medical staff is an important force in the fight against COVID-19. First-line emergency nurses have direct contact with confirmed patients in their work, participate in emergency treatment, complete nursing work and take care of their lives. Yet, even under strict protection, they are highly likely to be infected. Some previous studies have focused on the PTG of emergency nurses, but the research on their psychological coping and growth after being infected with COVID-19 need to be further explored. It is of interest and significance to understand how this representative group of nurses obtain PTG experience. Therefore, they were chosen as study subjects in the present study, and the phenomenological research method for qualitative research was applied to explore their experience and process of PTG so as to provide a new perspective and theoretical basis for psychological rehabilitation or intervention for medical staff who experienced traumatic events.

## Methods

### Study design

Phenomenological qualitative study and individual semi-structured interviews were used to explore the subjective feelings and experience of PTG of first-line emergency nurses infected with COVID-19, and to understand the positive factors affecting PTG. In simple terms, phenomenology seeks to describe the essence of a phenomenon by exploring it from the perspective of those who have experienced it. The goal of phenomenology is to describe the meaning of this experience—both in terms of what was experienced and how it was experienced (21). This method provides an in-depth perspective on the experiences of the participants (22), focuses on describing common experiences shared across a population (23).

### Participants and ethical considerations

First-line nurses in the emergency department of a third-level class-A hospital in Shanghai who participated in the treatment of COVID-19 patients and were infected with Omicron virus in April 2022 were included in the study. The method of purposeful sampling was used to select participants, which is used to identify and select informative cases related to the phenomenon of interest (24). The inclusion criteria were the following: (1) participated in the first-line emergency treatment of COVID-19 patients; (2) nucleic acid test positive for novel coronavirus infection; (3) informed consent and willingness to participate in this study; (4) qualified in verbal communication skills. Potential participants were contacted by email. Ethical approval was obtained from the Institutional Review Committee of Shanghai Tenth People's Hospital. The researchers explained the purpose and process of the study and ensured the confidentiality of the data. Personal interviews were scheduled with the consent of the participants. All the nurses participating in the study gave their written informed consent, and informed that they had the right to withdraw from the study at any time, without reason. To secure the participants' privacy and the confidentiality of their data, in place of their real names, code names were used throughout the text. Finally, 13 first-line emergency nurses with COVID-19 diagnosis were interviewed, all of whom volunteered to participate in the study and no one dropped out. General characteristics of participants are shown in Table 1.

**Table 1:** General characteristics of participants.

**Characteristics**	***N* (%) or mean (SD)**
**Sex**	
Male	3 (23.08)
Female	10 (76.92)
**Age**	26.33 (3.75)
**Length of employment**	3.92 (4.06)
**Education level**
Diploma	2 (15.39)
Baccalaureate degree	11 (84.61)
**Marital status**	
Married	2 (15.39)
Single	11 (84.61)
**Place of birth**	
Jiangsu province	2 (15.39)
Anhui province	4 (30.78)
Jilin province	1 (7.69)
Gansu province	1 (7.69)
Henan province	1 (7.69)
Hunan province	1 (7.69)
Sichuan province	1 (7.69)
Chongqing city	1 (7.69)
Shanghai city	1 (7.69)

### Data collection

Semi-structured in-depth interviews were conducted in June 2022 through face-to-face communication. A semi-structured interview guide was created to guide actual interviewers in gathering data. Table 2 shows the interview questions included in the guide, which were constructed on the basis of previous research (25–27). The guide would start the interview with encouraging questions. Participants shared their traumatic experiences and coping process, and these were explored until full understanding of emerging themes was achieved (28). If necessary, the interviewer would ask follow-up questions that were more in-depth and specific. All researchers had past experience in qualitative interview. Before the study began, the participants had no relationship with the researchers, i.e., they did not know each other from before. In addition, prior to data collection, two emergency nurses were pre-interviewed to ensure the clarity and identification of any potential problems. The pre-interview was considered as a test and was not included in the analysis. Although there is no established principle regarding the sample size in qualitative studies, data collection is considered finished when satisfactory data are gathered, no new information emerges anymore and when the same data start to appear (29). In following this norm, the sample size and domain size were estimated at the point of saturation (13 participants). With permission from all participants, interviews were recorded by the investigators. Each interview lasted 40–60 min and was conducted by two researchers and two research assistants. In addition to the interview, participants' responses were recorded, including non-verbal cues and body language during the interview. These were later transcribed verbatim and analyzed concurrently.

**Table 2:** The interview guideline: open questions.

**No**.	**Questions**
1	What were your experiences and feelings after being infected with COVID-19?
2	How did you cope with the difficulties and stress during this period?
3	What positive changes have you made?
4	After this infection, what do you think is the biggest change in yourself?
5	What other insights do you have?

### Data analysis

The audio recordings were transcribed verbatim and checked for accuracy by repeated listening within 24 h of the interviews. Data analysis was independently performed by two experienced researchers immediately after each interview. The Colaizzi's phenomenological seven-step method (30) was used for data analysis and to complete the extraction of themes and sub-themes. In this study, the seven-step process is illustrated in Table 3. If there were differences in opinion between researchers, they were discussed until a consensus was reached. The final transcribed data and extracted topics and subtopics were sent to the participants, all of whom agreed to be contacted again. Through feedback communication, it was ensured that the research results reflected the actual views of participants, and the results were not biased due to the subjective perception of the researchers.

**Table 3:** Colaizzi's seven-step process for qualitative data analysis.

**No**.	**Data analysis step**
1	All interviews were recorded and transcribed. Each transcript is carefully read several times.
2	Re-read, highlight, and extract meaningful statements directly related to the perspectives and experiences of first-line emergency nurses in PTG.
3	Formulate meanings from all significant statements.
4	Identify and organize the formulated meanings into theme clusters.
5	Describe the investigated phenomenon of PTG in the first-line emergency nurses exhaustively.
6	Recognize similar subthemes, identify the basic structure, and get the main themes.
7	Return to the participants to confirm the findings. The authors discussed their disagreements until a consensus was reached.

### Study rigor

To ensure the dependability of the study, the methods and the analyses used were described in detail. Credibility was ensured by a “blinded” approach to the materials by each researcher. Validity was ensured by continuous triangulation among researchers with regard to discordant texts. Peer information was used for verification. In addition, the data were re-evaluated by an expert in qualitative studies, who was not included in the study. Lastly, for transferability of the data, the sample and the data were described in detail.

## Results

**Figure 1:**
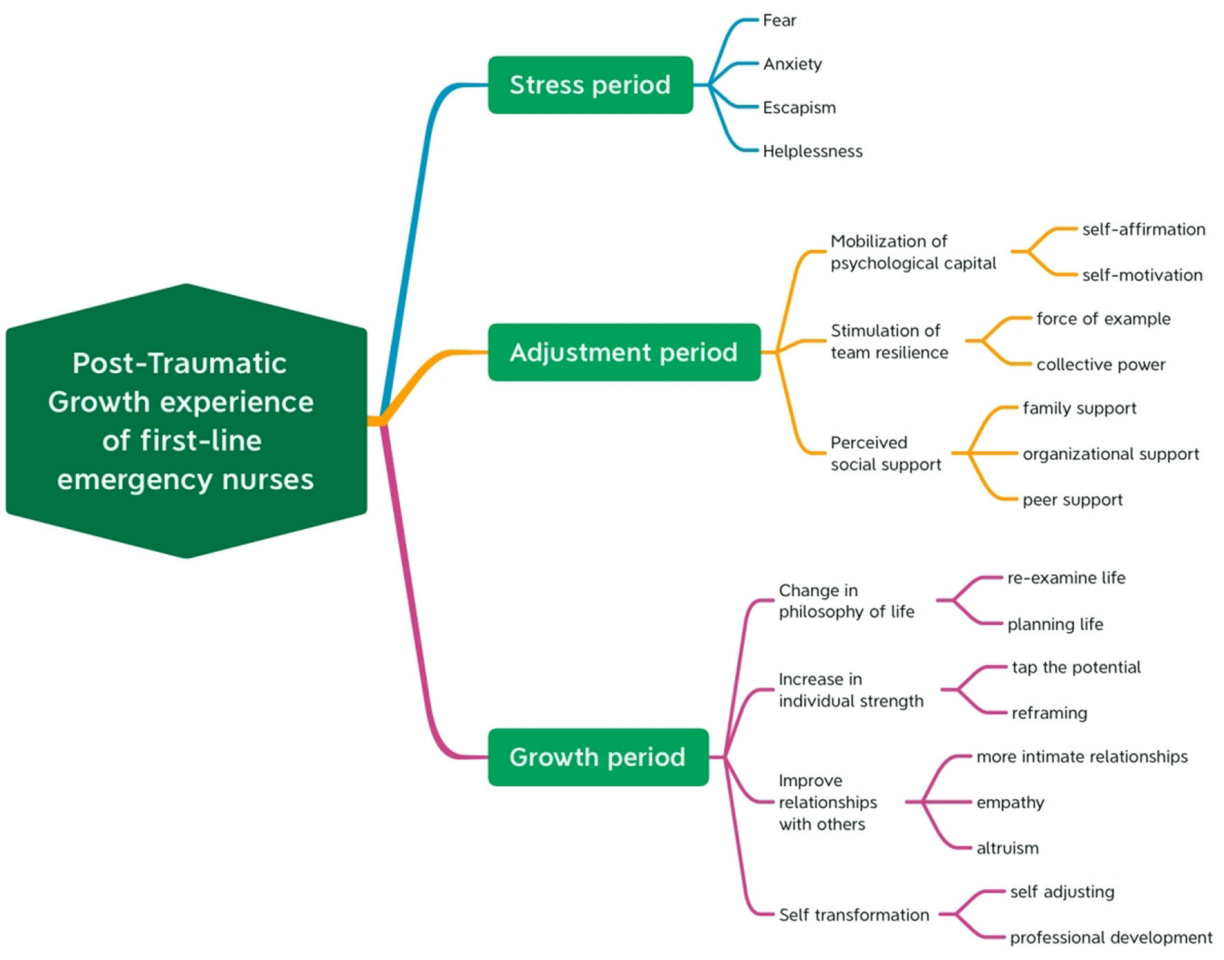
Themes and sub-themes categorized from data.

A total of 13 participants including 3 men and 10 women were included in the study. They were originally from nine different provinces or municipalities in China. Their average age was 26.33 years old and they had an average of 3.92 years of work experience. Their education level ranged from diplomas (*n* = 2) to baccalaureate degrees (*n* = 11). Most of the participants were unmarried (*n* = 11). All of them were infected with COVID-19 (*n* = 13). By using Colaizzi's methodology, three main themes and several sub-themes were identified. Themes were described and extracted from the experiences and insights of emergency nurses infected with COVID-19 at different stages during the fight against the virus. Three main themes were: (1) stress period; (2) adjustment period; (3) growth period. Themes and sub-themes categorized from the data are presented in Figure 1.

### Stress period

#### Fear

All participants expressed the fear of receiving a positive nucleic acid test notification. They expressed fear of COVID-19 symptoms, after effects, risk of transmission, and imminent isolation. “*I received a call from the hospital to inform me that I was infected. I suddenly felt overwhelmed, felt tightness in my chest and even felt that the air would be frozen..*.” (Participant 6). “*I was sent to the makeshift hospital in the middle of the night. I cannot describe the kind of fear I experienced... (shake)*” (Participant 2). With the onset of COVID-19 symptoms, physical pain and the presence of the virus in the body were felt, causing nurses to experience unprecedented fear. “*Fever, cough, muscle aches, and lack of energy. I was worried about developing pneumonia, and I was even more afraid of going to the makeshift hospital, because there are so many infected patients*” (Participant 10). “*My first symptoms included fever, cough, pain in my eyes, a laceration of my throat, up to the manubrium sternum, and chest tightness. I was particularly worried that I would have after-effects from the novel Coronavirus infection. I am still so young. I don't want any health-related issues..*.” (Participant 13).

#### Anxiety

While undergoing isolation and treatment, most participants were deeply worried and disturbed. One participant experienced these emotions as she was missing her family members. “*My hometown in Anhui, my parents have been urging me to go back. My family took this matter very seriously, and I had to show them all my meals at the isolation area by WeChat so that they could rest assured. They were so nervous that I could hardly hold back my tears when I would see them (choking). Their sad appearance made me more anxious and I missed them deeply*” (Participant 4). As symptoms change, nucleic acid test results have become a great matter of concern among these nurses. If the situation did not improve, they would fall into deeper anxiety, some of them even unable to rest normally. “*I had a cough for a month, accompanied by a low fever, and was extremely agitated. Colleagues who got infected around the same time as I did have already returned to work, but I still have not recovered, and I was worried about whether I had pneumonia and whether it would develop into tuberculosis (frowns). I have fallen into excessive restlessness and have to use sleeping pills to fall asleep*” (Participant 11). “*Colleagues infected around the same time got negative nucleic acid test results, while I was still positive. I was every day in a state of fear waiting to check the nucleic acid report on the phone*” (Participant 7).

#### Escapism

Most participants adopted escapism behavior toward possibility of being infected with COVID-19, which was unacceptable to them, while some felt stigmatized. “*I didn't want anyone to know I was infected, including my family, as I did not want them worrying about me*” (Participant 1). Stigma was often associated with social stereotypes, prejudice and discrimination, all of which have a negative impact on patients' social recovery. One participant thought that COVID-19 patients were loathed by society (31). “*I don't want to be labeled as COVID-19 patient*” (Participant 8). They did not even want to recall the experience. “*It would be nice to have the ability to erase that experience the way you format a computer file*” (Participant 2).

#### Helplessness

Some participants expressed feelings of helplessness about what they were going through. “*One day, a neighbor in our community was infected, and an ambulance came to take her to the isolation area. Someone took a video of her being picked up and posted the video in a group chat with the caption “The sheep (refers to patients diagnosed with COVID-19) has finally been picked up!” I was very sad at that moment; worrying about what kind of ridicule would I have to face in the future?*” (Participant 9). One participant described the disappointment of living in makeshift hospital caused by a sudden change of circumstances and restricted freedom. “*In the makeshift hospital, although you were given three meals a day, I can only move in the makeshift hospital, I really did not know what to do, I feel like every day there was wasted (sigh)*” (Participant 3).

### Adjustment period

#### Mobilization of psychological capital

Positive psychological capital is known to have an important role in improving employee's job performance (32). In the field of nursing, studies have shown that individuals with better psychological capital can face difficulties at work with confidence and optimism (33). Most nurses reported experiences of self-affirmation and self-motivation. “*I accepted the negative energy of the negative emotions and told myself that I can do it. Although some problems still exist, my soul is invigorated*” (Participant 5). “*Dr. Julie Noram, an American psychiatrist, once said, “There are people who tend to be pessimistic in their behavior, but still achieve remarkable results.” I think I might be a defensive pessimist (chuckle)*” (Participant 1).

#### Stimulation of team resilience

Team resilience is an important protective factor of individual resilience reorganization (34). Participants described comfort and encouragement among individuals in the same organization that helped them grow. “*Our infected colleagues who went to the isolation area together set up a WeChat group. Every day, we tracked improvement of symptoms and nucleic acid status of members in the group and comforted each other*” (Participant 6). “*Several of our infected colleagues huddled together for warmth and felt less terrible*” (Participant 2).

#### Perceived social support

External support from social, colleagues, and families can effectively help nurses improve their ability to adapt to stress and psychological adjustment. The supportive relationship's encouragement and care can help individuals maintain good psychological states during trauma recovery (35). All respondents expressed warm social support, including family support, organizational support and peer support. “*My mom and dad talk to me on video every day about my condition. I felt I needed to get better soon*” (Participant 7). Good social relations and social resources help individuals to obtain external support in adversity and reduce stress response. The support of team leaders helps build confidence in individuals, and their support is of guiding value. “*I clearly remember that when I went to the isolation area, the head nurse prepared a lot of materials for me, such as disinfection supplies, protective supplies, food, daily necessities, and a brand new trolley box. She asked about my symptoms every day, which was very touching*” (Participant 4). Support and care from first-line team members are very motivating and heartfelt, which also reflects team cohesion. “*When I was released, two colleagues who were just off their night shift helped me carry things to the hotel. I really appreciated it, as it helped me to feel lonely wandering alone in Shanghai*” (Participant 3).

### Growth period

#### Change in the philosophy of life

All participants mentioned a new understanding of life and the future after experiencing trauma. They would reexamine the meaning of life and re-plan their lives for the future. “*Nothing is better than a healthy life, and nothing is important as health*” (Participant 3). “*I will get better for myself and my family; I will spend more time with them, cherish every day, and enjoy the fun of life. After all, there are many meaningful things I still need to do*” (Participant 13).

#### Increase in individual strength

Resilience is the ability of individuals to make positive choices and rationally deal with stress. It is beneficial for lead individuals to reframe the non-adaptive state and activate their potential to defend against crisis so as to resolve difficulties (36). Most of the participants expressed their experience of exploring their strengths and reframing. “*Just like the principle of a pressure cooker, if I keep pressuring myself, I may lose control or my negativity may eventually erupt. Although it is hard, I believe I can deal with these problems better*” (Participant 1). “*I cannot turn back the time, and I still have a long way ahead of me. I will try to accept the reality, and continue on my way, as one can still grow in the face of adversities*” (Participant 9).

#### Improve relationships with others

All participants reported improved relationships with others, including closer relationships with family and friends. They are more empathetic and altruistic in PTG. “*When I saw my son at the gate of the community after I came back from isolation, I burst into tears and held him tightly in my arms*” (Participant 10). “*I felt closer to my best friend, who encouraged me to go share my feelings with him as we could go through them togethe*r” (Participant 12). Traumatic experiences of nurses can potentially affect how they treat patients, and empathy can improve relationships with patients. “*When I had the opportunity to experience the same type of pain my patients' experience, I could really feel and understand them better*” (Participant 4). Volunteering to help others can make one feel good and enhance the sense of worth. This triggers the release of endorphins, which help to reduce anxiety and contribute to good health (37). “*I'm more willing to help patients at work. It's really hard to be sick. Being a patient is a painful role*” (Participant 1).

#### Self-transformation

After traumatic events, most people tend to adjust to adversity and gradually recover to a state of health. People have abilities for self-healing and resilience, which tend to vary from person to person (38). All the nurses who participated expressed the process of self-transformation. “*I adjusted my mind and rose above myself in adversity (smile)*” (Participant 11). In addition to the psychological, one participant described an experience in which he took positive action to improve himself. “*I signed up for an online training course for emergency specialist nurses of Shanghai Nursing Association to keep myself busy and continue to work on myself (laughing)*” (Participant 9).

## Discussion

This study, carried out to reveal the process and influencing factors of post-traumatic growth among emergency nurses infected with COVID-19 was completed with 13 nurses. Emergency nurses experienced psychological changes before and after being infected with COVID-19, and these emotions were universal rather than special. Various internal and external factors continued to contribute to their recovery from traumatic events. Overall, participants described this process as a transition from negative to positive. The process and causes of PTG in this study agree with those presented in the literature (26, 39, 40).

Previous studies have focused on the psychological response of nurses to negative events, mostly in view of the anxiety, depression and other negative aspects (41, 42). In the research results, nurses experienced negative emotions as the main aspect. However, some recent studies suggested that PTG was common in nurses, so it was necessary to discuss the psychological process of nurses experiencing stress from the perspective of positive psychology. Most of the existing studies on PTG among nurses were completed by quantitative investigation, where traumatic events included war, earthquake disasters, secondary exposure to violence in hospital work, exposure to workplace violence, traumatic childbirth, caring for a terminally ill patient or experiencing the death of a patient (43–46), while qualitative research has a significant effect on the profound exploration of personal inner feelings, especially for such a serious stress event as COVID-19 infection. According to the results of this study, all first-line emergency nurses involved in the study experienced PTG after being infected with COVID-19. PTG did not immediately occur at first, but it resulted from effective adaptation and improvement. Individuals, groups and society came into play as different factors in this process.

The concept of post-traumatic growth, which was first proposed by Americans scholar Tedeschi and Calhoun (18), is interpreted by some researchers as finding benefits, psychological thriving, adversary growth, perceived benefits, stress-related growth, and similar. It refers to the positive change experienced by an individual after struggling with a challenging life crisis, which makes the individual grow and surpass the original level at least in one aspect (18). The initial research subjects used in the PTG studies were college students with traumatic experience, and later the research focus shifted to acute traumatic events. Patients with chronic and severe diseases such as cancer, AIDS and leukemia have become the main research subjects in the past decade (47). Meanwhile, related studies have begun to focus on the PTG experience of caregivers of diseased patients (48). Studying the PTG of individuals in public health emergencies is an innovative approach. Different identities, traumatic events and cultures may lead to different fields and specific connotations of individuals' PTG. The nurses we interviewed here were not only caregivers of COVID-19 patients, but also infected patients, a very special group.

Previous studies have shown that PTG often coexists with significant psychological distress (43, 45). The results of this study showed that at the early stage of COVID-19 infection, emergency nurses were in a strongly negative psychological state, which researchers named “Stress period.” Fear, helplessness, anxiety and escapism were the most common psychological manifestations of the stress period. Clinical nurses were the main force in disaster relief, facing many pressure sources. The unpredictability of the SARS-CoV-2 Omicron Subvariants, side effects and sequelae of infection, and the fear of receiving isolation treatment were all factors that usually cause great psychological stress in the infected person. Experienced nurses were more calm and less affected by stressful events, and traumatic experiences could have existed in their previous careers (49–51). Among the interviewees, many emergency nurses were young nurses without adequate disaster relief experience, which might imply that their psychological quality was relatively weak, and further influence their attitudes toward work and their perceptions of careers. In addition, infected nurses with high self-esteem did not want to be cared and treated by their families or society as COVID-19 patients (52). In traditional Chinese culture, some people interpret having an unfortunate illness as a form of stigma, so they prefer not to share their condition with anyone. Stigma is often associated with social stereotypes, prejudice and discrimination, which negatively impact patients' social recovery (31). When someone has a condition that might be interpreted as shameful and unpleasant, people tend to reject and “mark” them, thus increasing stigma. Accordingly, society as a whole should make more efforts to eliminate the public's discrimination and prejudice against COVID-19 patients.

After a period of strong stress, the infected emergency nurses gradually adapted to their status and the role of patients and entered the “adjustment period.” Some positive factors helped them make a positive change, manifested as an improvement in resilience. Resilience implies the ability to bounce back or easily recover when confronted by adversity, trauma, misfortune, or change (53). The key point of resilience is adapting to various environments. Researchers defined resilience in nursing as a measure of a nurse's ability to cope with stressors and mental health threats, where resilient people are emotionally calmer while dealing with catastrophic situations (54). For nurses, resilience is one factor that helps reduce their stress levels and increase endurance. Nurses mobilize psychological capital through self-affirmation and self-motivation. Positive psychological strength and excellent psychological qualities improve adaptability (38). In addition, Chinese culture emphasizes the importance of tenacious character. People with tenacious character tend to have a strong will; they are fearless and able to bear hardships. These characteristics help individuals stick to endure and actively seek ways to overcome difficulties. Positive and effective coping strategies enable individuals to show higher self-efficacy when encountering setbacks or difficulties and mobilize available resources to solve problems, which is an important factor for realizing PTG (55). In the emergency medical teams, colleagues having to cope with the same types of accident encourage and support each other, thus forming a strong organizational cohesion, which is important for improving team resilience. High personal and team resilience are positive factors associated with improved career development. Social support refers to the social resources provided by formal or informal support groups that are perceived subjectively and/or received objectively by individuals (56). External support comes from society, organization, colleagues and family. In our study, nurses appreciated receiving material or spiritual support and help under adverse circumstances. Other studies have reported that for medical staff, social support has a direct positive predictive effect on PTG, and can also indirectly promote PTG through psychological resilience (57).

The “growth period” theme extracted from the interviews refers to a change in philosophy of life, increase in individual strength, improved relationships with others, and self-transformation, all of which reflect several aspects of post-traumatic growth of nurses after COVID-19 infection. According, Self-determination Theory (SDT), when certain needs are destroyed after a traumatic event, individuals still take action to restore themselves to a state of wellbeing (58). The traumatic event of being infected with COVID-19 leads individuals to reframe in a non-adaptive state, revealing their previously unexposed potential (59), such as dealing with problems, learning from the environment, and adjust mindset. Nurses get well and subsequently acquire a new concept of life, which is regarded as growth. They have more experience and a better mentality to review and face the traumatic events that may occur later on in their future life. Nurses diagnosed with COVID-19 received meticulous and professional care from their nursing colleagues as patients, which allowed them to gain a deeper understanding of the profession and recognize the significance of nursing work. They were also under greater expectations to attain professional advancement through efforts. The experience of being helped and cared for strengthened their bonds with family, friends and colleagues. Moreover, due to the role transformation, nurses infected with COVID-19 gained the ability to more deeply understand the helplessness of patients and the importance of humanistic care. Their empathy and altruism were enhanced, thus guiding them to think and solve problems from the perspective of patients and providing warm, humanistic care for patients.

Traumatic events were detrimental to nurses' physical and mental health and professional development. Studies have shown that clinical nurses' turnover intention has a significant internal relationship with the traumatic events they have experienced (60). As it is not possible to avoid traumatic events, nursing managers should consider how to help nurses cope with traumatic events and achieve PTG, which is related to the construction of the nursing team and the quality of clinical nursing. Self-disclosure is the act of revealing private information to others (61). The PTG theory holds that self-disclosure can promote an individual's ability to achieve PTG (62). Therefore, after traumatic events, nursing managers should pay more attention to the psychological state of nurses in their daily work, actively communicate with them, and encourage them to express emotions. A positive, purposeful rumination should be cultivated. Deliberate rumination refers to the adaptive cognitive process of paying attention to the negative emotions or experiences caused by traumatic events, and actively, consciously, and purposefully explaining traumatic events, seeking meaning, and exploring inner feelings (63), which is beneficial for new nurses in improving their resilience and post-traumatic growth (64). On-going education and guidance may protect infected nurses from absorbing or internalizing unmanageable emotions which may lead to compassion fatigue and also help them to gain a deeper understanding of their communication and interactions with patients (17, 65). For example, mindfulness, workshops, situational simulation exercise, peer support and other methods can be used to train nurses to better deal with traumatic events (66). We further encourage medical institutions to set up support organizations, specifically for the mental health of medical staff, form the trauma informed culture as, which could greatly facilitate developing and implementing team PTG intervention strategies.

## Limitations

This study did not consider certain factors that could affect an individual's ability of PTG, such as gender, religious beliefs and any previous personal experiences with traumatic events. Moreover, because this was a qualitative study, the results were not replicable, as the subjects, experiences and contexts were all unique. Lastly, the study was performed in a single clinical setting, which further hindered the generalization of the results. Replication of this study in different clinical settings, with larger samples, and with samples from different cultures could help to generate more diverse findings. If the study had included focus group interviews, this could provide more in-depth themes in nurses' experience.

## Conclusion

First-line emergency nurses infected with COVID-19 are a special group that should be paid more attention to. Investigating how they achieve their psychological adjustment and growth in the case of severe trauma can provide valuable references for nursing management and future education. Different aspects of a traumatic event are associated with significant changes in the mind and behavior of affected individuals. Individuals, organizations, and societies contribute to their PTG. However, there are many aspects that can be improved to achieve PTG. As suggested in this study, society, hospital and nursing managers should pay more attention to the PTG of nurses and establish supportive PTG strategies to make up for the lack of individual coping ability, which will benefit the retention rate and career development of nurses.

## Data availability statement

The original contributions presented in the study are included in the article/supplementary material, further inquiries can be directed to the corresponding authors.

## Author contributions

JJ and PH: conception, design, and revising the article critically for intellectual content. JJ, PH, XH, YL, and HS: acquisition of data. JJ, PH, LZ, and XD: analysis, interpretation of data, and drafting the article. All authors contributed to the article and approved the submitted version.

## Funding

This work was supported by the Shanghai Shenkang Hospital Development Center Clinical Science and Technology Innovation Project (SHDC12021611) and Shanghai Medical Union Theory Research Key Project (2022YGL10).

## Conflict of interest

The authors declare that the research was conducted in the absence of any commercial or financial relationships that could be construed as a potential conflict of interest.

## Publisher's note

All claims expressed in this article are solely those of the authors and do not necessarily represent those of their affiliated organizations, or those of the publisher, the editors and the reviewers. Any product that may be evaluated in this article, or claim that may be made by its manufacturer, is not guaranteed or endorsed by the publisher.
